# Polymer-Based Instructive Scaffolds for Endodontic Regeneration

**DOI:** 10.3390/ma12152347

**Published:** 2019-07-24

**Authors:** Naimah Zein, Ezeddine Harmouch, Jean-Christophe Lutz, Gabriel Fernandez De Grado, Sabine Kuchler-Bopp, François Clauss, Damien Offner, Guoqiang Hua, Nadia Benkirane-Jessel, Florence Fioretti

**Affiliations:** 1 French National Institute of Health and Medical Research (INSERM), Regenerative Nanomedicine, UMR 1260, FMTS, 67085 Strasbourg, France; 2 Faculté de Médecine de Strasbourg, Strasbourg, Université de Strasbourg, 67000 Strasbourg, France; 3 Pôle de Chirurgie Maxillo-Faciale et Stomatologie, Hôpitaux Universitaires de Strasbourg, 67000 Strasbourg, France; 4 Faculté de Chirurgie Dentaire de Strasbourg, Université de Strasbourg, 67000 Strasbourg, France; 5 Hôpitaux Universitaires de Strasbourg, Pôle de Médecine et Chirurgie Bucco-Dentaires, 67000 Strasbourg, France

**Keywords:** scaffolds, polymers, endodontics regeneration, dental pulp, dental stem cells, active biomolecules, hydrogels, nanofibers

## Abstract

The challenge of endodontic regeneration is modulated by clinical conditions which determine five kinds of tissue requirements: pulp connective-tissue formation, dentin formation, revascularization, reinnervation and radicular edification. Polymer scaffolds constitute keystone of the different endodontic regenerative strategies. Indeed, scaffolds are crucial for carrying active molecules and competent cells which optimize the regeneration. Hydrogels are very beneficial for controlling viscosity and porosity of endodontic scaffolds. The nanofibrous and microporous scaffolds mimicking extracellular matrix are also of great interest for promoting dentin-pulp formation. Two main types of polymer scaffolds are highlighted: collagen and fibrin. Collagen scaffolds which are similar to native pulp tissue, are adequate for pulp connective tissue formation. Functionnalization by active biomolecules as BMP, SDF-1, G-CSF enhances their properties. Fibrin or PRF scaffolds present the advantage of promoting stem cell differentiation and concomitant revascularisation. The choice of the type of polymers (polypeptide, PCL, chitosan) can depend on its ability to deliver the active biomolecule or to build as suitable hydrogel as possible. Since 2010s, proposals to associate different types of polymers in a same scaffold have emerged for adding advantages or for offsetting a disadvantage of a polymer. Further works would study the synergetic effects of different innovative polymers composition.

## 1. Regenerative Endodontics

### 1.1. Generalities

Regenerative endodontics aims to replace damaged endodontic structures: pulp connective tissue with its vascularization, its innervation and its peripheral dentin [1,2]. The modality of conventional endodontic treatment involves removal of all damaged pulp tissues from teeth and their replacement by inert material after adequate disinfection. For many years, this drastic treatment has been considered as the unique treatment without alternative strategies [1].

In the 1960s, Obstby proposed an approach for regenerating dental pulp tissue by promoting bleeding into the root canal [3]. This procedure had been then forgotten for over twenty years. Afterwards, the concept of recreating a vascular network in the root canal or ‘’revascularization’’ substantiated [4,5]. Initial clinical case reports of revascularization concerned regeneration of immature teeth affected by pulp necrosis and apical periodontitis [6,7,8].

**Figure 1:**
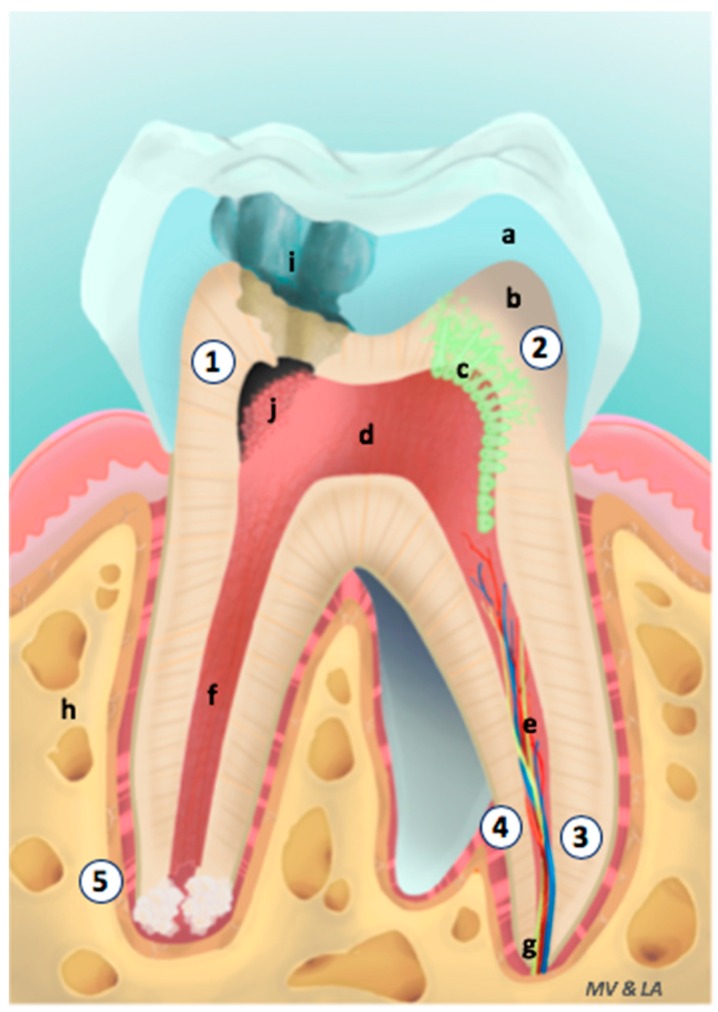
Levels of endodontic regeneration. 1; Pulp Connective tissue Formation, 2; Dentin Formation, 3; Revascularization, 4; Reinnervation, 5; Radicular Edification. a: Enamel, b: Dentin, c: Odontoblasts, d: Pulp, e: Blood vessels and nerves, f: Root canal, g: Apex, h: Bone, i: Lesion, j: Pulp fibroblasts.

For more than a decade following first case reports, different dental pulp regeneration strategies have been proposed inspired by advances of regenerative medicine. Polymeric scaffolds are the heart of these innovant strategies [9]. Indeed, the probability of creating a new functional tissue pulp by the unique injection of stem cells without matrix or any active biomolecules is very low [2,9,10]. Scaffolds provide a solid environment for adhesion, proliferation and differentiation of competent cells and are able to orchestrate drug delivery of active biomolecules. Thus, biodegradable 3D implantable or injectable scaffolds which are able to carry stem cells and to deliver growth factors, have become the most suitable method for dental pulp regeneration [9]. Several scaffolds differing by their structure and nature have been developed and reported with interesting findings. Once implanted, these scaffolds must constitute adequate pro-regenerative microenvironment, which is able to attract healthy resident cells to the lesion, notably stem cells [9,11,12,13,14,15]. Scaffolds can target five different levels of regeneration: (i) pulp connective-tissue formation, (ii) dentin formation, (iii) revascularization, (iv) reinnervation and (v) radicular edification (Figure 1).

### 1.2. Stem Cells

Studies of dental pulp regeneration complex involving stem cell sources have been conducted for long time. Concerning dentin formation, mesenchymal stem cells (MSCs) of pulp origin have the biggest potential to differentiate into odontoblast-like cells [16,17,18]. In 2004, it was reported that MSCs from dental pulp, periodontal ligament (PDL), and bone marrow generate the same tissues as their origins which supported the idea that dental pulp regeneration would require pulpal MSCs present in the root canal [19]. Moreover, dental pulp stem cells (DPSCs), stem cells from human exfoliated deciduous teeth (SHED) and stem cells of apical papilla (SCAPs) are considered as potential cell sources for regeneration of the dental pulp complex [20,21].

It is known that the quality and availability of cells from dental pulp tissue decline strongly with age so much that investigations are needed in order to find alternative sources of cells [1]. Generation of induced pluripotent stem cells (iPSCs) constituted a revolution in regenerative medicine. It consists on a reprogramming of somatic or differentiated cells, to a pluripotent state by overexpression of several defined transcription factors [22,23,24]. Patient specific iPSCs are analogous to embryonic stem cells because they can give rise to all cell lines in the body. In addition, they can circumvent the clinical barrier of immunological rejection or ethical concepts [23]. Recently, a protocol has been reported to induce neural crest-like cells (NCLCs) by the differentiation of murine iPSCs (miPSCs) *in vitro* [25]. These NCLCs have the potential to differentiate into dental mesenchymal cells, such as odontoblasts, upon their co-culture with mouse dental epithelial cells. These interesting results have paved the way for a strong potential of iPSCs in future regenerative dental research.

### 1.3. Biomolecules

**Figure 2:**
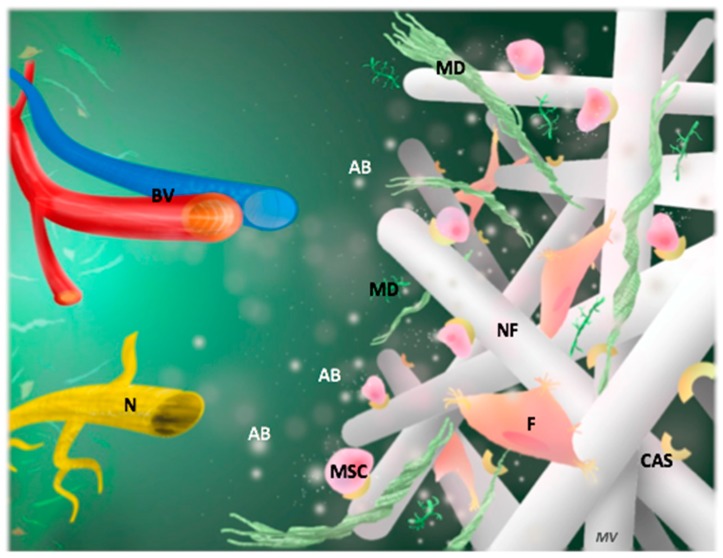
Polymer instructive scaffold for endodontic regeneration. Incorporation of active biomolecules inside the scaffold allows orchestration of tissue regeneration and attraction of resident cells according to ‘’cell homing process’’. Stem cells cultured in the scaffold optimize the pulp regeneration, notably matrix deposition. AB; Active biomolecules (white rounds), BV; Blood vessels, CAS; Cellular adhesion site, F; Fibroblasts, MD; Matrix deposition, MSC; Mesenchymal stem cells, N; Nerves, NF; Nanofiber.

Biomolecules (BM) that are secreted from demineralized dentin matrix or exogenously delivered are found to participate importantly in pulp regeneration by forming favorable microenvironments [26,27]. They are known to recruit endogenous cells by chemotactic effects and induce differentiation of recruited cells to initiate dentin-pulp regeneration, called ‘’cell homing process” (Figure 2). Studies with biomolecules, either singularly or in combination, have shown various aspects of its function.

Transforming growth factor-β (TGF-β) family is a key family of growth factors, that have been identified in the dentin matrix. This family comprises several factors, such as TGF-β, bone morphogenetic protein (BMP), anti-Mullerian hormone (AMH) and growth differentiation factors (GDFs). TGF-β1 is involved in primary odontoblastic differentiation and promote tertiary dentin generation with the regulation of dentin extracellular matrix synthesis, proliferation, growth, differentiation and apoptosis of cells [26]. TGF-β3 is involved in the induction of ectopic mineralization in dental pulp during tooth germ development of fetal mouse [28]. TGF-β molecules could act as regulator in the initiation of functional differentiation of odontoblasts [29]. BMP-2 stimulates the differentiation of dental pulp stem cells into odontoblasts *in vitro* and *in vivo*, and it can induce dentin sialophosphoprotein (DSPP) expression to enhance the angiogenic potential of DPSCs [30]. In addition, BMP-2 increases alkaline phosphatase activity and stimulates reparative dentin formation [31].

Blood-derived growth factors like vascular endothelial growth factor (VEGF), platelet-derived growth factor (PDGF), TGF-β, fibroblast growth factor (FGF), and insulin growth factor (IGF) exist in blood clots. They are produced by clinical bleeding induction [3,4,5]. VEGF is considered as a dominant signaling protein, it is involved in lymphangiogenesis, vasculogenesis and angiogenesis [32]. It promotes blood vessel formation enhancing neovascularization [33]. PDGF is an important mitogen for cells of mesenchymal origin [34]. Moreover, PDGF promotes angiogenesis and regulates the process of odontoblastic differentiation and acting synergically with other growth factors [35].

## 2. Structure of Polymer Scaffolds

### 2.1. Properties

For clinical dental application, scaffolds must be easily introduced in narrow spaces. The initial color and the color of the scaffold after its degradation must be compatible with aesthetic function of teeth. Pulp is a soft connective tissue protected mechanically by mineralized tissue: dentin and enamel (Figure 1). It means that the mechanical properties of scaffold are not critical. The porosity of endodontic regenerative scaffold is crucial. The size and the density of pores must be perfectly controlled. One hundred μm is the minimum pore size for tissue regeneration. The porosity allows migration and proliferation of cells, transport of nutriments and active molecules and elimination of wastes [36,37].

### 2.2. Hydrogels

Hydrogels of natural and synthetic polymers are appropriate materials for dental pulp regeneration, because they constitute injectable scaffolds. Reliable viscosity and flexibility are assured by their water content [9]. Poly-Ethylene Glycol Maleate Citrate (PEGMC) hydrogel is an injectable drug delivery vehicle, it was developed for direct pulp capping and showed interesting results with a proper cell viability and control of the incorporated calcium hydroxide [38].

The quantity and the size of pores which are critical characteristics for hydrogels can be controlled. Thus, the optimal porosity of poly-L-lactic acid (PLLA) scaffold hydrogels can be obtained to induce proliferation and differentiation of DPSCs [39,40]. However, the long fabrication process, especially the self-assembled peptide hydrogels, as well as the limited incorporation of nanofibers are the major inconvenience of the use of hydrogels [9].

In 2008, Galler et al. demonstrated that a peptide-amphiphile (PA) self-assembling hydrogel presented an easy way for endodontic insertion and promoted DPSCs and SHEDs cell proliferation and dental pulp formation [41]. Later, peptide hydrogels were proposed as suitable drug delivery system for VEGF, TGF-β1 and FGF-1. They induced an odontoblast-like cell differentiation and pulp-like tissue formation [42]. In addition, a self-assembling multidomain peptide (MDP) hydrogel with DPSCs cells released several growth factors and induced pulp-like tissue formation [43]. Moreover, peptide hydrogel (PuramatrixTMno) self-assembling scaffolds are composite hydrogels used for dental-pulp regeneration and induce DPSCs proliferation and differentiation [44].

### 2.3. Nanofibers

Nano-fibrous and microporous membranes are very useful technologies to build pro-regenerative mimic extracellular matrix [45] (Figure 2). Different natural and synthetic polymer matrices are developed, by electrospinning, with nano-fibers of diameters tightly close to the size of collagen nano-fibers (from 50 to 500 nm). The electrospun randomized nano-fiber connection and the created micropores (diameter less than 100 μm) mimic the pattern of the connective tissue matrix [9,46,47]. Electrospun matrices of poly(ε-caprolactone) (PCL) present favorable scaffolds for connective tissue regeneration [48,49]. The capacity of these synthetic electrospun matrices to carry active biomolecules, so to be ‘’functionalized’’ is very beneficial.

Different strategies are used for this functionalization of the electrospun nano-fibers: surface graft polymerization, plasma or wet chemical treatment and incorporation into the polymer solution to electrospin [50]. The advantage of this last technique named ‘’co-axial electrospinning’’ is an envelopment of the bioactive molecule inside the nano-fibers for a prolonged action. Antibiotics and antioxidants are incorporated into PCL nano-fibers [51]. The association of electrospraying and electrospinning is useful to functionalize nano-fibers during their production. Nano-fibers of poly(methyl methacrylate) (PMMA) are functionalized by alpha acid lipoic or sodium fluorides [52].

The mineralization of PCL scaffolds is strongly attractive for dentin tissue regeneration by inducing the growth and odontogenic differentiation of human dental pulp cells (hDPCs) [53]. The incorporation of mesoporous bioactive nanoparticles in the nano-fibrous PCL-gelatin matrices enhances this odontogenic differentiation of hDPC [54]. Nano-fibrous gelatin/magnesium phosphate scaffolds exhibit the release of metallic ions that enhances dentin regeneration by human pulp stem cells (hPSCs) [55].

The functionalization of nano-fibrous PCL scaffolds by neural growth factor (NGF) induces innervation ascending from the root to the coronal part of the pulp *in vivo*. It is a particularly important step for dental pulp regeneration. This innervation allows tooth functionality and tissue homeostasis, such as dentinal sensitivity, odontoblast function, masticatory forces [56]. These electrospun nano-fibrous membranes can also help in the disinfection by incorporation of antibiotics. Nanocomposite scaffolds containing polydioxanone II (PDS II) with metronidazole (MET) or ciprofloxacin (CIP) present a crucial antimicrobial activity against *Enterococcus faecalis* (*Ef*) and *Porphyromonas gingivalis* (*Pj*) and induce dental pulp formation [57].

## 3. Composition of the Polymer Scaffolds

### 3.1. Collagen

Recombinant or animal-derived collagens, especially collagen (type I), are the most useful biomaterials for tissue engineering, drug delivery models and cosmetic surgery [11]. Scaffolds made by collagen are used in several systems, either in fibrillary native forms or in denaturized forms like sponges, plugs, sheets, and pellets [11].

In 1994, Nakashima et al. showed that collagen scaffolds combined with BMP-2 and 4 induced osteodentin and pulp-tissue formation [58]. More than ten years later, a study using collagen scaffold with ceramic powder (CP) and the dentin matrix protein 1 (DMP-1) with DPSCs cells demonstrated that this scaffold induced new pulp-tissue formation with an adequate organization [59].

Interesting findings were observed after autologous transplantation of collagen scaffold seeded with pulp CD31- side-population (SP) cells or CD105^+^ cells and containing stromal-cell-derived factor-1 (SDF-1) into the root canals of dogs. Complete pulp regeneration with strong vascularization and innervation were also obtained [60,61,62].

Besides, collagen scaffold containing granulocyte-colony-stimulating factor (G-CSF) promoted mobilization, high proliferation rates and differentiation of DPSCs cells [63].

Studies conducted by Iohara showed later that the use of mobilized DPSCs with G-CSF in a collagen scaffold induced *in vivo*, a pulp tissue regeneration, a coronal dentin formation and DPSCs differentiation in dog model [64,65]. Recently, a study also demonstrated that the use of collagen scaffolds had many beneficial effects in the seeding, proliferation and differentiation of hDPCs cells [66] (Table 1).

### 3.2. Gelatin

Gelatin is derived from the lysis of collagen; it is also appropriate for dental pulp regeneration. Ishimatsu et al. have reported that a gelatin hydrogel with incorporation of fibroblast growth factor-2 (FGF-2), was suitable for the colonization of dental pulp cells and revascularisation [67]. In 2017, Gelatin methacryloyl (GelMA) hydrogels built with adequate physical and mechanical properties, enhanced the odontoblast-like cells (OD21) viability and proliferation. In addition, these regenerative GelMA hydrogels seeded by endothelial colony forming cells (ECFCs) promoted also the formation of endothelial monolayers. It seemed to be an effective strategy for succeeding both pulp-formation and revascularization [68].

### 3.3. Fibrin

This naturel polymer promotes the initiation of wound healing inside connective tissues and therefore, can play a very important role for promoting first steps of pulp connective tissue formation. Fibrin scaffolds are crucial for differentiation of stem cells and for its hemostatic properties. In 2011, Galler et al. showed that the use of fibrin gel scaffold with polyethylene glycol (PEG) in multiple stem cells induced proliferation, pulp tissue formation, and an easy endodontic insertion [69].

### 3.4. Platelet-Rich Fibrin (PRF)

Platelet-rich fibrin (PRF) is a generation of platelet concentrate that contains multiple growth factors and exhibits cell differentiation properties, as well as having the capacity of degrading quickly. PRF is in the same time, a scaffold for dental pulp cell adhesion and migration, and a strategic source of growth factors [70]. The transplantation of DPSCs supported by PRF scaffolds onto the canal could help as potential therapy for regenerative endodontics, pulp vitality, or revascularization [70].

In 2016, a method for getting a PRF scaffold for endodontic regeneration was reported. It consisted in adding a hDPC suspension before centrifugation of blood. It showed that the obtained PRF could play a synergistic role in the formation of odontoblast cells with dentin matrix [71]. In addition, mineral trioxide aggregate (MTA) had also a synergic effect with PRF on dental pulp cells for promoting revascularization [72]. However, a comparison study between Platelet-rich plasma (PRP) and PRF showed that PRP was better than PRF in peripheral wound healing when used in regenerative clinical procedures [73].

### 3.5. Alginate

Alginate is a naturel polymer extracted from seaweed. Alginate hydrogels are developed to have a large range of applicability as biomaterials. They are known to be used as model of extracellular matrices for cell culture. They are precious for tissue engineering due to their adaptable stiffness, which allows variations of mechanical features. They are also very convenient for drug delivery. In 2002, Dobie et al. developed an alginate hydrogel with TGF-β1 which induced odontoblast-like cell differentiation [74]. Different alginate hydrogels seeded by stem cells of the apical papilla (SCAP) were proposed. Their composition influenced their microstructure, their mechanical and surface properties so much that it modulated considerably the viability of these stem cells [75]. The composition of Alginate scaffold containing nano-hydroxyapatite promoted mineralization and differentiation of human DPSCs. They can promote pulp as dentin formation [76].

### 3.6. Chitosan

Chitosan is a natural cationic polymer which presents a big interest for forming hydrogel. Its hydrophilic nature with ability of degradation by human enzymes results in important biocompatibility and biodegradability. The possibility of including nanofibers inside chitosan based hydrogels is also beneficial. Hydrogels based in chitosan offer a lot of potentials for regenerative medicine. Due to their capacities to induce mineralisation, they can support not only pulp connective tissue formation but also for dentin formation. The polycationic characteristic of chitosan gives them hemostatic and antimicrobial properties.

Chitosan scaffold with β-tricalcium phosphate promoted an increase in the expression of demineralization markers, such as alkaline phosphatase (ALP) and osteopontin (OPN). It induced also dentin-tissue formation and vascularization by human periodontal ligament cells (HPLCs) [77]. Chitosan-calcium-aluminate scaffold (CH-AlCa) delivering 1α,25-dihydroxyvitamin D3 (1α,25VD) increases migration and odontoblastic differentiation of dental pulp cells (DPCs) [78]. Chitosan scaffolds containing silver and bioactive glass promote also odontogenic differentiation of DPCs without impacting their proliferation. Decrease of inflammation and concomitant inhibition of streptococcus mutans and Lactobacillus casei growth are also obtained [79].

### 3.7. Poly-L-lysine Dendrigraft (PDGL)

In case of pulpits, reduction of inflammation is required before regeneration. A pro-regenerative anti-inflammatory polymer scaffold made by Poly-L-Lysine Dendrigraft (DGL), α-Melanocyte Stimulating Hormone (alpha MSH) and Poly-Glutamic Acid (PGA) was proposed [80], in which PGA-alpha-MSH promoted the decrease of inflammation of pulp connective tissue acting on fibroblasts, monocytes and macrophages. DGLG4-PGA-α-MSH nano-reservoirs induce the initiation of the regeneration of pulp connective tissue by providing adhesion and proliferation of pulp fibroblasts. The long-term action of these polymer nano-assemblies built by layer-by-layer nanotechnology may be needed to prevent inflammation aggravation and to let the regeneration of the tissue occur [81].

### 3.8. Polymers of Lactic Acid

The advantage of these synthetic polymers is to be easily modified for controlling their capacity of degradation. This property allows the scaffold to be a temporary supporting structure for growing cells and tissues [82]. Poly-L-Lactic acid (PLLA) and Poly-L-lactic-coglycolic acid (PLGA) are interesting synthetic polymers of lactic acid. PLLA is attractive for its slow degradation rate. When DPSCs cells were seeded on PLLA scaffolds, their attachment, proliferation and differentiation were enhanced [83]. Poly-L-lactic-coglycolic acid (PLGA) is also greatly interesting for mesenchymal regeneration. A modulation of mechanical properties of the scaffold is possible due to the versatility of its structure. PLGA microsphere scaffolds induce proliferation and differentiation of HDPCs into odontoblast-like cells. Bilayered PLGA scaffolds have been proposed able to induce a layer-specific dentinogenic differentiation of dental pulp stem cells (DPSCs) in vitro [84].

### 3.9. Composite Polymer Scaffolds

Collagen composite scaffold was built with poly(L-lactide-co-ε-caprolactone) (PLCL) and hyaluronic acid (HA). This combination presents a very high porosity and enables adhesion and growth of DPSCs cells, as well as proliferation and pulp-tissue formation [85].

Besides, rhCollagen PuramatrixTM hydrogel is a very promissing peptide hydrogel-based nanofibrous scaffold. Puramatrix refering to a self-assembling peptide hydrogel, is composed of a 16-mer peptide in an aqueous solution. After its interaction with some physiological conditions, it can polymerize and form a biodegradable nanofiber hydrogel scaffold. Puramatrix with SHED cells promote the generation of a pulp-like tissue when injected into full-length root canals. This strategy could help in the completion of the root formation in necrotic immature teeth [44]. In addition, porous collagen/chitosan scaffold releasing BMP-7 gene, induced the differentiation of DPSCs into odontoblasts-like cells *in vitro* and *in vivo* [45].

Composite scaffolds that combined gelatin and PCL with nano-hydroxyapatite promoted the differentiation of DPSCs into odontoblast-like cells *in vitro* and *in vivo* [86]. However, poly (D, L-lactide-coglycolide) (PLGA) with gelatin scaffold enhanced endodontic regeneration by simulation of extracellular matrix environments of stem cells [87]. Inflammatory reactions initiated by degradation products were decreased by the introduction of gelatin to PLGA-based scaffolds [88].

Composite polymeric scaffold that use PLGA and PLLA together, combined with DOX, induced pulp-tissue formation and inhibited bacterial growth for a long duration [89]. Poly-D, L-lactide scaffold combined with glycolide promoted differentiation of DPSCs and SCAPs cells, and induced pulp-like tissue formation with vascularity, as well as a dentin-like structure [90] (Table 2).

## 4. Conclusions

The challenge of endodontic regeneration is modulated by clinical conditions such as age of patient, immaturity of teeth, inflammatory and infection states. They determine the quantity of pulp resident healthy cells, the volume of pulp tissue to regenerate, the apical closure of teeth, the reinnervation and the revascularisation to obtain. Different promising regenerative strategies have been proposed to meet this challenge. The polymer scaffolds constitute the keystone of the strategy. Indeed, the simple injection of competent cells inside the tooth is poorly regenerative. Likewise, active biomolecules are required to promote the colonisation of cells and their matrix deposition. The incorporation of active biomolecules inside the scaffold is crucial for their delivery inside the injured tissues and so for their action to the competent cells. Thus, different polymer scaffolds carrying both competent cells and active biomolecules for endodontic regeneration have shown interesting results.

The viscosity and porosity of hydrogels easily controlled are very beneficial for endodontic applications. The nanofibrous and microporous scaffolds built by electrospinning mimicking extra-cellular matrix (ECM) are of great interest for promoting dentin-pulp formation. Two main types of polymer scaffolds for endodontic regeneration are highlighted: collagen and fibrin. Collagen scaffolds which are similar to native pulp tissue, are adequate for pulp connective tissue formation. Functionnalization by active biomolecules as BMP, SDF-1 and G-CSF, enhances their properties. Fibrin or PRF scaffolds present the advantage of promoting stem cell differentiation and concomitant revascularisation. Besides, PRF is naturally rich in growth factors. Some interesting proposals were made also with classic pro-regenerative polymers as chitosan, alginate and PCL. Other works chose the type of polymer according to its ability to deliver the active biomolecule or to build a biocompatible hydrogel with an as adequate as possible viscosity and porosity. Since 2010, proposals to associate polymers in a unique scaffold have emerged. The association of different types of polymers in a same scaffold aims to add different advantages or to offset a disadvantage of a polymer. Thus, the composite polymer scaffolds are more likely to achieve the different levels of the complicated endodontic regeneration. Further works would be to follow the synergetic effects between different polymer compositions and the added active therapeutics and cells.

## Figures and Tables

**Table 1:** Main instructive polymer scaffolds for different levels of endodontic regeneration.

Scaffold	Method	Associated Tissue Engineering Strategy		Regeneration Level *					Findings	Reference
**Peptide-amphiphile (PA) hydrogel self-assembling**	*In vitro*	-	DPSCs & SHEDs human	1					Easy for endodontic insertion Stem cell proliferation	Galler et al., 2008 [41].
**Peptide hydrogel**	*In vivo*	VEGF, TGF-β1 & FGF-1	DPSCs human	1	2	3			Release of VEGF, TGF-β1 and FGF-1 Odontoblast- like cell differentiation Pulp-like tissue formation	Galler et al., 2011 [42].
**Peptide hydrogel multidomain peptides (MDP) self-assembling**	*In vitro*	FGF, TGF-β1 &VEGF	DPSCs human	1		3			Pulp-like tissue formation	Galler et al., 2012 [43].
**PCL**	*In vivo*	Neural growth factor	mouse					5	Promotion of Innervation in a model of bioengineered tooth	Eap et al., 2014 [56].
**Polydioxanone II (PDS II)**	*In vitro*	MET or CIP	hDPSCs Human	1					Release MET or CIP Antimicribial activity against Ef and Pj	Bottino et al., 2013 [57].
**Collagen**	*In vivo*	BMP-2 and 4 & TGF-β1	Dog	1	2				BMP-2 and 4 induce osteodentin formation if combined with collagen matrix	Nakashima, 1994 [58].
	*In vivo*	CP & DMP-1	DPSCs human	1					New pulp-like tissue formation and organization human	Prescott et al., 2008 [59].
	*In vivo*	SDF-1	Dog pulp CD 105+, CD31 SP cells	1					Complete pulp-like tissue regeneration	Nakashima & lohara 2011 [60].
	*In vivo*	SDF-1	Dog pulp CD105^+^ cells	1		3	4		Complete pulp-like tissue regeneration Vascularization and innervation	Iohara et al., 2011 [61].
	*In vivo*	SDF-1	Dog pulp, BM, Adipose CD31 SP cells	1		3	4		Complete pulp-like tissue regeneration Vascularization and innervation	Ishizaka et al., 2012 [62].
	*In vivo*	G-CSF	hDPSCs human	1	2				Pulp-like tissue formation Differentiation of hDPSCs	Murakami et al., 2013 [63].
	*In vivo*	G-CSF	Dog mobilized DPSCs	1	2				Complete pulp-like tissue regeneration Coronal dentin formation in root canal	Iohara et al., 2013 [64].
	*In vivo*	G-CSF	Dog mobilized DPSCs	1					Differentiation of DPSCs Less volume of regenerated pulp-like tissue in aged dogs compared with that in young dog	Iohara et al., 2014 [65].
	*In vitro*	-	hDPCs	1	2				Beneficial effects on proliferation and differentiation of hDPCs	Kwon et al., 2017 [66].
**Gelatin hydrogel**	*In vitro*	FGF-2	Rat dental pulp	1		3			Release of FGF-2 Induces the invasion of dental pulp cells and vessels	Ishimatsu et al., 2009 [67].
**Methacryloyl GelMA hydrogel**	*In vitro*		OD21 ECFCs mouse	1		3			Cell viability, spreading and proliferation Simple and effective strategy for engineering of pre-vascularized dental pulp constructs	Athirasala et al., 2017 [68].
**Fibrin gel**	*In vivo*	PEG	DPSCs, SHEDs, PDLSCs BMSSCs human	1					All types of dental stem cells proliferated Easy for endodontic insertion	Galler et al., 2011 [69].
**Platelet-rich fibrin (PRF)**	*In vitro*	GFs	DPSC dog	1		3			Serve as a potential therapy in regenerative endodontics	Chen et al., 2015 [70].
	*In vitro*	GFs	hDPCs		2				Released the maximum quantity of growth factors	He et al., 2016 [71].
	*In vitro*	MTA	hDPCs		2				With MTA has a synergistic effects on odontoblastic differentiation of hDPCs	Woo et al., 2016 [72].
**PRF or Platelet—rich plasma (PRP)**	*Clinical*	-	human	1		3			PRP was better than PRF in peripheral wound healing when used in regenerative procedures	Shivashankar et al., 2017 [73].
**Alginate hydrogel**	*In vitro*	TGF-β1	human	1	2				Release of TGF-β1 Odontoblast-like cell differentiation	Dobie et al., 2002 [74].
**Alginate hydrogel**	*In vitro*	-	SCAPs	1					SCAPs proliferation	Lambricht et al., 2014 [75].
**Alginate**	*In vitro*	-	DPSCs human	1	2				Dental pulp mineralization Differentiation od DPSCs	Sancilio et al., 2018 [76].
**Chitosan**	*In vitro*	β-tricalcium phosphate	HPLCs human		2	3			Upregulated expressions of ALP and OPN	Liao et al., 2010 [77].
**Chitosan**	*In vitro*	1α,25-dihydroxyvitamin D3 (1α,25VD) Calcium-aluminate	DPCs human	1	2				Increased odontoblastic phenotype expression Cell migration	Bordini et al., 2019 [78].
**Chitosan**	*In vitro*	Silver Bioactive glass	DPCs	1	2				Decrease of inflammation Odontogenic differentiation of DPCs Inhibition of *Streptococcus mutans* and *Lactobacillus casei* growth	Zhu et al., 2019 [79].
**Poly-L-lysine**	*In vitro*	α-MSH	Rat-human	1					Poly-L-lysine (Dendrigraft) is favorable for colonization of pulp fibroblasts and to the delivery of anti-inflammatory hormone	Fioretti et al., 2010 [80]. Fioretti et al., 2011 [81].
**Poly(L-lactic acid) PLLA**	*In vitro* *In vivo*	-	DPSCs human	1					Attachment, proliferation and differentiation of DPSCs	Wang et al., 2011 [82].
**PLGA**	*In vitro*	-	DPCs human	1	2				Odontoblastic differentiation and proliferation	Zou et al., 2016 [83].
**PLGA**	*In vitro*	-	DPSCs	1					Differentiation of DPSCs	Gangolli et al., 2019 [84].

* Regeneration Levels; 1: Pulp Connective-tissue Formation, 2: Dentin Formation, 3: Revascularization, 4: Reinnervation, 5: Radicular Edification. Abbreviations: α-MSH: α-Melanocyte Stimulating Hormone, ALP: alkaline phosphatase, BM: bone marrow, BMP: bone morphogenetic protein, CIP: Ciprofloxacin, CP: ceramic powder, DMP-1: dentin matrix protein 1, DPSCs: dental pulp stem cells, ECFCs: endothelial colony forming cells, Ef: enterococcus faecalis, FGF-2: fibroblast growth factor 2, G-CSF: granulocyte colony-stimulating factor, GF: growth factor, hDPCs: human dental pulp cells, hDPSCs: human dental pulp stem cells, HPLCs: human periodontal ligament cells, MET: metronidazole, MTA: mineral trioxide aggregate, OD21: odontoblast-like cells, OPN: osteopontin, Pj: porphyromonas gingivalis, SDF-1: stromal-cell-derived factor-1, SHEDs: stem cells from human exfoliated deciduous teeth, SP: side-population, TGF-β1: transforming growth factor β 1, VEGF: vascular endothelial growth factor.

**Table 2:** Composite instructive polymer scaffolds for different levels of endodontic regeneration.

Scaffold	Method	Associated Tissue Engineering Strategy		Regeneration Level *			Findings	Reference
**Peptide hydrogel (Puramatrix)** **self-assembling**	*In vitro*	-	DPSCs human	1			DPSC survival, proliferation and differentiation	Cavalcanti et al., 2013 [44].
**Collagen** **Chitosan**	*In vivo*	BMP-7	DPSCs human animals		2		Release of BMP-7 gene DPSC differentiation into odontoblasts-like cells in vitro and in vivo	Albuquerque et al., 2014 [45].
**Collagen** **Poly(L-lactide-co-ε-caprolactone)**	*In vitro*	HA	DPSCs human	1			DPSC differentiation and proliferation	Akkouch et al., 2013 [85].
**rhCollagen** **peptide hydrogel (Puramatrix^TM^)**	*In vivo*	-	SHEDs human		2		SHED injected into full-length human root canals differentiate into functional odontoblasts	Rosa et al., 2013 [86].
**Gelatin** **poly(ε-caprolactone) (PCL)**	*In vitro*	nHA	DPSCs human		2		DPSC differentiation toward an odontoblast-like cells in vitro and in vivo	Yang et al., 2010 [87].
**Poly(lactic-co-glycolic acid) (PLGA)**	*In vitro*	GFs	DPSCs dog	1		3	PLGA/gelatin electrospun sheet made up a microenvironment for tooth root generation	Chen et al., 2015 [88].
**PLGA** **Poly(L-lactid acid) (PLLA)**	*In vitro*	DOX	-	1			Release of DOX Inhibition of bacterial growth for a prolonged duration	Feng et al., 2010 [89].
**Poly-D,L-lactide** **Glycolide**	*In vitro*	-	DPSCs & SCAPs human	1	2	3	Pulp-like tissue formation with vascularity and dentin-like structure	Huang et al., 2010 [90].

* Regeneration Levels; 1: Pulp Connective-tissue Formation, 2: Dentin Formation, 3: Revascularization. Abbreviations: BMP: bone morphogenetic protein, BMSSCs: bone marrow stromal stem cells, DOX: doxycycline, DPSCs: dental pulp stem cells, GF: growth factor, HA: hyaluronic acid, nHA: nano-hydroxyapatite, PEG: polyethylene glycol, PDLSCs: periodontal ligament stem cells, rhCollagen: recombinant human collagen, SCAPs: stem cells from root apical papilla, SHEDs: stem cells from human exfoliated deciduous teeth.
